# Toxicity and Pharmacogenomic Biomarkers in Breast Cancer Chemotherapy

**DOI:** 10.3389/fphar.2020.00445

**Published:** 2020-04-15

**Authors:** Zeina N. Al-Mahayri, George P. Patrinos, Bassam R. Ali

**Affiliations:** ^1^ Department of Pathology, College of Medicine and Health Sciences, United Arab Emirates University, Al-Ain, United Arab Emirates; ^2^ Department of Pharmacy, School of Health Sciences, University of Patras, Patras, Greece; ^3^ Zayed Center for Health Sciences, United Arab Emirates University, Al-Ain, United Arab Emirates; ^4^ Department of Genetics and Genomics, College of Medicine and Health Sciences, United Arab Emirates University, Al-Ain, United Arab Emirates

**Keywords:** breast cancer, chemotherapy, toxicity, pharmacogenomics, side effects

## Abstract

Breast cancer (BC) is one of the most prevalent types of cancer worldwide with high morbidity and mortality rates. Treatment modalities include systemic therapy, in which chemotherapy is a major component in many cases. Several chemotherapeutic agents are used in combinations or as single agents with many adverse events occurring in variable frequencies. These events can be a significant barrier in completing the treatment regimens. Germline genomic variants are thought of as potential determinants in chemotherapy response and the development of side effects. Some pharmacogenomic studies were designed to explore germline variants that can be used as biomarkers for predicting developing toxicity or adverse events during chemotherapy in BC. In this review, we reassess and summarize the major findings of pharmacogenomic studies of chemotherapy toxicity during BC management. In addition, deficiencies hampering utilizing these findings and the potential targets of future research are emphasized. Main insufficiencies in toxicity pharmacogenomics studies originate from study design, sample limitations, heterogeneity of selected genes, variants, and toxicity definitions. With the advent of high throughput genotyping techniques, researchers are expected to explore the identified as well as the potential genetic biomarkers of toxicity and efficacy to improve BC management. However, to achieve this, the limitations of previous work should be evaluated and avoided to reach more conclusive and translatable evidence for personalizing BC chemotherapy.

## Introduction

Breast cancer (BC) is the second most prevalent type of cancer worldwide after lung cancer. In women, it is the most common type of tumor and the leading cause of cancer-related deaths. Around 2.1 million women in 2018, were newly diagnosed with the disease and more than 600,000 women died of it world-wide (Bray et al., 2018). Due to the continued high morbidity and mortality rates, a considerable body of research is dedicated to improving outcomes of BC treatment and management.

Treatment of BC involves three main modalities: surgery, radiation, and drug therapy. Different institutions use several protocols, and various guidelines were developed. However, there are only marginal differences between these protocols, according to a comparison between 17 clinical practice guidelines used in the USA, Canada, Australia, UK, and Germany. Breast-conserving surgery is a standard recommendation by all protocols, though the staging and the appropriate procedure differs between different institutions. The definition of adequate surgical margins is another point of a discrepancy between different guidelines. Similarly, radiotherapy following breast-conserving surgery is recommended by all guidelines, while the definition of high-risk cases that necessitate radiotherapy following mastectomy is inconsistent (Wolters et al., 2012).

Drug therapies used for breast cancer are classically classified into three categories: 1) endocrine or hormonal therapy, 2) targeted therapies, including anti HER2, and 3) chemotherapy. Diverse combinations of these classes of drugs are given as either an adjuvant or neoadjuvant regimens. The adjuvant therapy follows the primary treatment by surgery with or without radiation to decrease the risk of distant recurrence in an approach that proved to improve outcomes and survival rates (Anampa et al., 2015; Carels et al., 2016). In contrast, neo-adjuvant regimens are given before surgery to downsize or downstage the tumor in the cases of locally advanced or large tumors (Joseph et al., 2012; Carels et al., 2016).

The phenotypic subtypes, known as intrinsic subtypes, are among the main predictive factors affecting the choice between different systemic therapeutic agents. The expression of hormonal receptors including estrogen receptors (ER), and progesterone receptors (PR), and the absence or presence of human epidermal growth factor 2 (HER2) receptors, known as luminal A or B subtypes, in the tumor tissue determine the suitability of the patient for hormonal or targeted therapy. Hormone therapy includes selective estrogen receptor modulators such as tamoxifen or aromatase inhibitors. While the high expression of HER2 receptors alone without the hormonal receptors, known as Her-2 enriched subtype, makes the anti-Her2 therapies such as trastuzumab with or without chemotherapy, the treatment of choice. The positive expression of both types of receptors by the tumor tissue makes the combination of all hormonal, anti-Her2, and chemotherapy an appropriate choice. In contrast, having triple-negative (ER-ve, PR-ve, and HER2-ve) tumor tissue, known as the basal subtype, leaves the patient with no systemic therapy options other than chemotherapy (Anampa et al., 2015). The proliferation-biomarker Ki67 has been recently used as an additional predictive indicator during the systemic therapy decision making, but with contradictory evidence (Ács et al., 2017).

**Figure 1:**
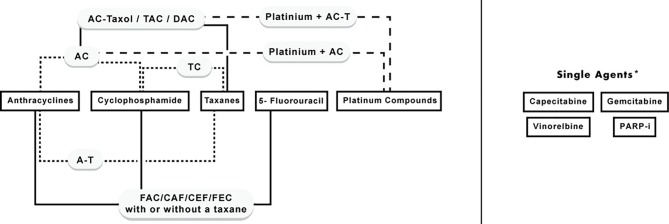
Classes of chemotherapy agents used in BC and their combinations. *Single agents: include agents that are commonly used as single agents in advanced or recurrent cases, however, they can be given in combination with first-line chemotherapy agents.

Chemotherapy is an essential component in many cases of BC, and different classes of cytotoxic agents are being used. Indeed, it is the backbone of systemic therapy in metastatic BC, and indispensable in many subtypes of early BC; specifically, for luminal B, Her-2 enriched, and triple-negative cases. Nevertheless, some cases of early luminal A (ER-positive and HER-2 negative) with high tumor burden (e.g., three or more lymph nodes are involved) benefit from chemotherapy (Twelves et al., 2016; Cardoso et al., 2019). The main chemotherapy classes used are anthracyclines, anti-microtubules (taxanes), alkylating agents (cyclophosphamide), antimetabolites (5-fluorouracil, capecitabine), platinum compounds (cisplatin), and others. These drugs are usually given in multiple-drug regimens, which proved to be superior to single agents in terms of efficacy and safety (González-Neira, 2012). However, in cases of advanced BC, some chemotherapies are given as single agents. The classes and main combinations of chemotherapeutics used in the management of BC treatment are summarized in **Figure 1**.

Although their well-established benefit on overall survival, chemotherapy side effects are a major concern that affects treatment outcome. The current approach to counteract chemotherapy side effects is to administer lower doses, to postpone the chemotherapy dose until the acute effect subside, or to add other drugs to treat them. These approaches can risk the patient's benefits from treatment and might compromise disease control. Nevertheless, the agents given to counteract chemotherapy toxicities have their associated side effects; for example, constipation resulting from overuse of anti-diarrhea agents to treat chemotherapy-induced diarrhea (Nurgali et al., 2018). More seriously, the administration of granulocyte colony-stimulating factor (G-CSF) to counteract chemotherapy-induced neutropenia doubles the low risk of developing secondary leukemia following chemotherapy (Azim et al., 2011). Unfortunately, many BC patients refuse to start or to complete their recommended treatment due to side effects (Puts et al., 2015; Hamelinck et al., 2016). Accordingly, there is an urgent need to control these events, given that survival rates decrease from 84.7 to 46.2% in patients who refuse treatment (Joseph et al., 2012).

Outcomes of BC systematic treatment in terms of response and the occurrence of adverse events are heterogeneous even in patients with a similar grade, stage, and subtype. This variation is contributed by many factors, including the patient's age, menopausal state, alcohol use, smoking, diet, and other medications. However, genetic factors have also been shown as important determinants in the treatment outcomes (González-Neira, 2012).

BC patients, like other types of cancer patients, have two genomes; their constitutional “germline” genome and their tumor tissue “somatic” genome(s). The latter harbors the constitutional variations plus the acquired changes that have been either tumor-causing (i.e., oncogenic drivers) or developed during tumorigenesis as passenger mutations. Even within the tumor, there is a degree of genomic heterogeneity between the various lineages. Genomic variants linked to a pathological process or drug response or a drug target, are referred to as biomarkers.

A massive body of research has focused on investigating somatic biomarkers in breast cancer tissue and comparing these between different patients, which resulted in the introduction of more targeted therapies. For example, the FDA recently approved *PIK3CA* inhibitor, alpelisib, for patients with hormone receptor-positive, and HER2 negative tumors that harbor *PIK3CA* mutations (André et al., 2019).

In contrast, although they are important, germline biomarkers attracted less research attention. Investigating germline DNA variants is not expected to lead to designing new drugs. However, these variants are amongst the determinants of traditional treatment response and toxicity. Biotransformation is a limiting factor in the activation of prodrugs and the elimination of almost all drugs. In the case of breast cancer, some studies evaluated the effect of variants in genes related to systematic therapies biotransformation and transportation with limited conclusions and clinical utility (Hlavac et al., 2018).

In this review, we will focus on toxicity and adverse events associated with systemic chemotherapy of BC and the germline biomarkers of these events investigated through what is known as “pharmacogenomic studies of toxicity.” We intended here to evaluate and summarize the contribution of germline genome variants in chemotherapy adverse events in breast cancer, highlighting the deficiencies in this area and the potential targets. Hormonal therapies and targeted therapies are beyond the scope of the current review as they have been previously covered in various reviews (Goetz et al., 2008; Del Re et al., 2012; Yiannakopoulou, 2012; Sanchez-Spitman et al., 2019). Also, pharmacogenomic predictors of response have been extensively reviewed elsewhere (Lee and McLeod, 2011; Sacco and Grech, 2015) and will not be included here. In contrast, up to our knowledge, there are no published reviews that cover all the commonly used chemotherapeutic toxicity pharmacogenomic predictors, specifically in BC. Herein, we will summarize the mechanisms and usage of different classes of cytotoxic agents in breast cancer and their associated adverse events. Then we will be describing with details the accumulated evidence on pharmacogenomic predictors of these adverse events.

## Chemotherapy in Breast Cancer and Their Associated Toxicity and Side Effects

**Figure 2:**
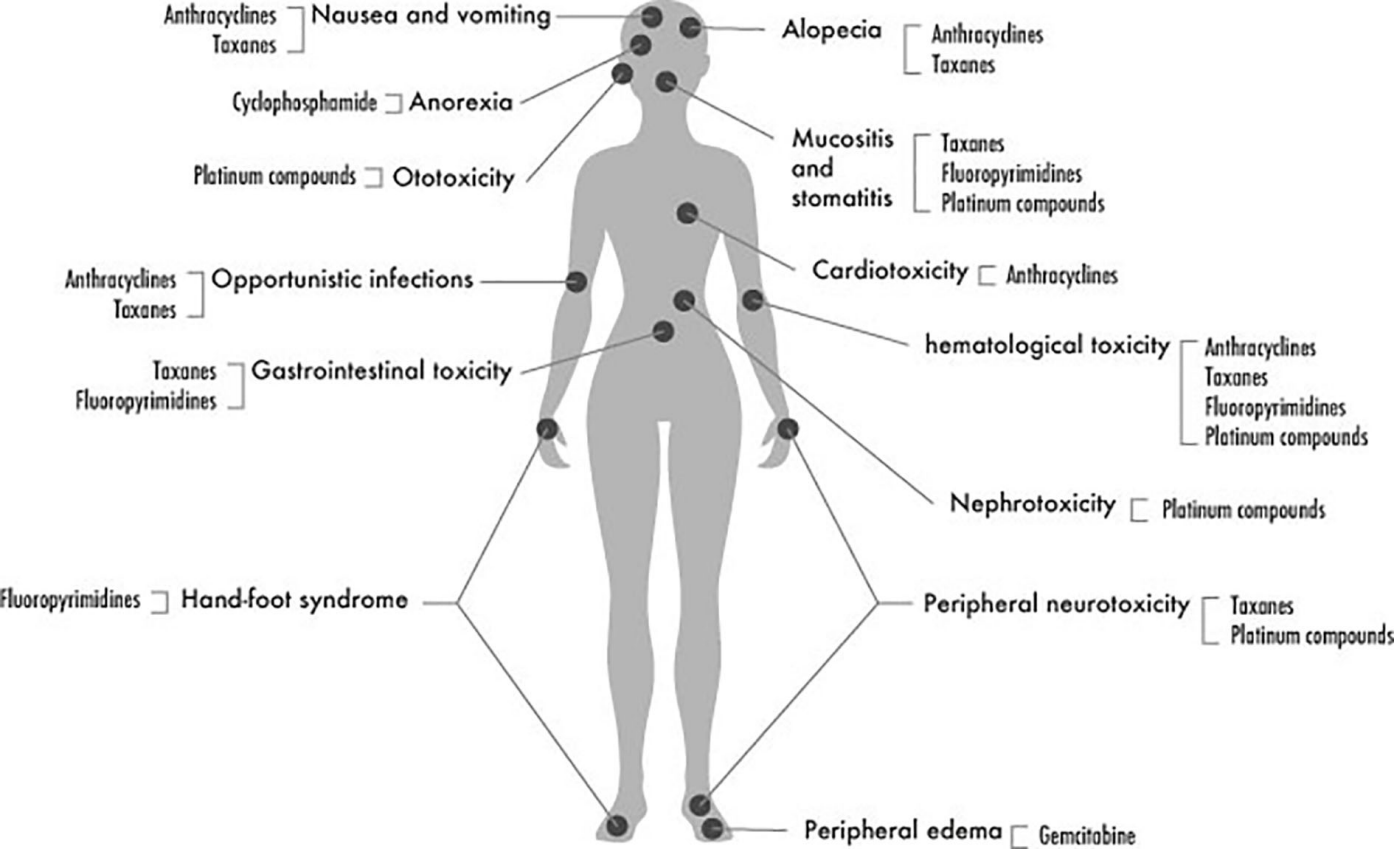
Most common side effects of chemotherapy in BC.

The most common adverse events and toxicities encountered during chemotherapy of BC are illustrated in **Figure 2** and summarized in the following sections:

### Anthracyclines

Anthracyclines, including doxorubicin, epirubicin, daunorubicin, and idarubicin, are considered among the most powerful chemotherapeutics used in many types of solid tumors and leukemia. They form the backbone of chemotherapy regimens used in BC in the neoadjuvant or adjuvant settings. Anthracyclines are frequently used in combination with cyclophosphamide (Sacco and Grech, 2015). This combination, known as AC, replaced the older combination of cyclophosphamide, methotrexate, and 5-fluorouracil (CMF) after proving its superiority for BC cases in terms of survival and reducing recurrence (Bray et al., 2010; Jamieson and Boddy, 2011; Leong et al., 2017).

Anthracycline-associated adverse events are considered as a significant limiting factor in utilizing these powerful cytotoxic agents. These events include mainly cardiotoxicity that might occur as acute toxicity manifested by arrhythmias or depressed ejection fraction, particularly in the left ventricle (LVEF) or might be chronic that develops years after the anthracycline use (Schneider et al., 2017). In addition to their cardiac effects, hematological toxicity, gastrointestinal toxicity, and febrile neutropenia events are all among the dose-limiting side effects of anthracyclines.

### Cyclophosphamide

Cyclophosphamide is a DNA alkylating agent that is considered a cornerstone in the chemotherapy of most types of tumors (Pinto et al., 2009) and a component of almost all combinations used in breast cancer chemotherapy (Emadi et al., 2009). The recommended doses of cyclophosphamide in breast cancer are usually considered as intermediate doses that have few side effects. However, using these doses for a long time may induce chronic toxicities. The main cyclophosphamide adverse event that requires monitoring is hematological toxicity. Nausea and vomiting are other common side effects, besides reversible alopecia (Emadi et al., 2009).

### Taxanes

Taxanes, including paclitaxel and its semi-synthetic analog docetaxel, are potent cytotoxic agents that act as microtubule stabilizers. They exert their cytotoxic action through binding to tubulin molecules leading to an increase in microtubule polymerization, a suppression in microtubules dynamics, and eventually mitotic block and cell death (Jordan and Wilson, 2004). Adding taxanes to breast cancer chemotherapy regimens in the neoadjuvant, and the adjuvant setting was found to significantly enhance the pathological response and overall survival, respectively (Hertz et al., 2014). A meta-analysis that included 28,853 patients from 24 trials concluded that the chemotherapy regimen composed of doxorubicin, cyclophosphamide and followed by paclitaxel (known as AC-T) is the most effective adjuvant therapy in early breast cancer (Fujii et al., 2015).

Taxanes' side effects, including mainly hematological suppression and neurotoxicity, are considered major dose-limiting factors. Hematological toxicity is common in docetaxel, while peripheral neuropathy occurs more with paclitaxel. Gastrointestinal toxicity and hypersensitivity reactions are some of the other reported adverse events (Bosó et al., 2014; Frederiks et al., 2015).

Taxane induced neuropathy (TIN), manifested by neuropathic pain, paresthesia, and in some cases sever neurotoxicity leading to loss of function, is a severe adverse event that might lead to treatment disruption (Kus et al., 2016). It has been reported that 5 to 30% of breast cancer patients suffer from grade 3 or 4 TIN, while these rates increase to around 50 to 80% of breast cancer patients when considering grade 2 neuropathy (Abraham et al., 2014). The given dose, treatment schedule, age, comorbidities, preceding treatment regimens, and alcohol use are among the factors that determine TIN occurrence (Kus et al., 2016). Moreover, some studies investigated the genetic factors related to TIN (Frederiks et al., 2015).

### 5-Fluorouracil (5-FU)

5-Fluorouracil (5-FU) has been one of the oldest chemotherapies used in breast cancer that is still currently utilized. It was introduced within the CMF (cyclophosphamide, methotrexate, 5-FU) combination in the seventies of the last century during the early stages of introducing systematic management of BC to enhance the earlier local approaches. The effectiveness of this combination was evaluated three decades later and shown significant prevention from BC-related deaths in rates up to 30% during that 30 years interval (Bonadonna et al., 2005). Later evidence (Group (EBCTCG) EBCTC, 2012) of the superiority of CAF (oral cyclophosphamide with Intravenous [IV] doxorubicin and 5-FU) or FAC (IV 5-FU, doxorubicin, and cyclophosphamide) over CMF made the preceding two regimens more favored by oncology institutions.

5-FU containing regimens are usually associated with hematological toxicity including anemia, leukopenia, and thrombocytopenia. Stomatitis and hyperpigmentation are other commonly reported toxicities (Shajahan et al., 2015).

### Capecitabine

Capecitabine is a third-generation fluoropyrimidine that is approved for pretreated metastatic breast cancer (Syn et al., 2016). Capecitabine is commonly used after anthracycline/taxane-based therapies failure during palliative therapy. Several trials evaluated adding capecitabine to the conventional cytotoxic regimens in the neoadjuvant and adjuvant setting but with limited benefit. However, this was not the case for triple-negative BC patients, were capecitabine found to be exceptionally improving overall survival and disease-free survival (Caparica et al., 2019).

The most common side effect of capecitabine is the hand-foot syndrome (HFS), which is reported in 43 to 71% of patients using it as a monotherapy. It was suggested that the predisposing factors for HFS might differ from those related to other capecitabine side effects, namely hematological and digestive toxicity (Yap et al., 2017).

### Platinum Compounds

Platinum compounds, including carboplatin and cisplatin, exert their cytotoxic effect by introducing DNA crosslinks, which halt the replication fork (Heijink et al., 2019). These agents have been for a long time an unfavorable option in breast cancer due to the availability of other less toxic agents that are also easier in administration (González-Neira, 2012). More recent evidence about the superior benefit of these agents over other cytotoxic drugs in triple-negative BC patients with *BRCA*1/2 mutations brought back these old drugs under focus (Heijink et al., 2019).

The toxicity of platinum compounds is a major constraint during their use. Specifically, acute and chronic neurotoxicity usually leads to dose reduction or treatment cessation (McQuade et al., 2018).

### Other Chemotherapy Agents

#### Gemcitabine

Gemcitabine is an antimetabolite that is utilized in advanced BC. Controversial outcomes were retrieved from studies that evaluated the benefit of adding gemcitabine to treatment regimens or using it as a single agent in advanced BC. One recent metanalysis indicated that using gemcitabine in combination therapies demonstrated favorable outcomes in advanced, however, with considerable associated hematological toxicity (Xie et al., 2018).

#### Vinorelbine

Vinorelbine is another option in first-line treatment or pretreated metastatic BC. Hematological toxicity again is the most common adverse event associated with its use, followed by nausea and vomiting (Cybulska-Stopa et al., 2013).

#### Poly-ADP-Ribose Polymerase Inhibitors (PARPi)

Poly-ADP-Ribose Polymerase Inhibitors (PARPi) inhibit the activity of PARP as a DNA damage sensor and consequently halt this pathway of DNA repair. Two agents from this class, namely olaparib and talazoparib, have been recently approved for advanced cases of *BRCA1*/2 positive BC (McCann et al., 2019).

#### TDM1

TDM1, a drug-antibody conjugate that is formed from the combination of trastuzumab, the anti-HER2 agent, and DM1, the cytotoxic anti-microtubule, also known as emtansine. TDM1 was found effective in reducing recurrence and mortality rates by 50% more than trastuzumab alone, among HER2+ BC patients who have residual invasive disease following finishing the recommended neoadjuvant therapy (von Minckwitz et al., 2019).

## Pharmacogenomic Studies of Chemotherapy Toxicity in Breast Cancer: Review and Critical Evaluation

Pharmacogenomic studies, in general, follow one of two major designs; candidate-gene approach and genome-wide association studies (GWAS). In the former, genes that are involved in the metabolism, transport, excretion, or targets of the drug of interest are explored for biomarkers that show statistically significant associations with the studied outcome (i.e., response or adverse events or both). Otherwise, genes might be selected because of their speculated involvement in the outcome of interest rather than drug pharmacokinetics. On the other hand, in GWAS, variants covering the whole genome are investigated against a specific outcome of interest in a presumption-free approach. In the following sections, we summarize and critically evaluate the evidence of pharmacogenomics (PGx) for adverse events of individual chemotherapeutic agents or combination regimens.

### Anthracyclines

#### PGx Studies of AIC

Anthracycline induced cardiotoxicity (AIC) is one of the most studied toxicities in cancer. Left-sided radiation therapy in some cases of left-sided BC and the use of trastuzumab, which is known for its cardiotoxic effects, in Her-2 positive cases are two other cardiotoxicity disposing-factors that might not be considered in big studies recruiting patients with different types of tumors (Vulsteke et al., 2015). Gender is considered another risk or protective factor in the case of cardiotoxicity. The effect of gender on susceptibility to AIC is still controversial; however, it should be considered while investigating the genetic factors that might be associated with this adverse event (Meiners et al., 2018). Accordingly, there is a need to explore AIC in female BC patients exclusively.

Five candidate-gene studies with a total of 1,779 females BC only participants were screened for polymorphisms in selected genes (Vulsteke et al., 2015; Hertz et al., 2016; Reinbolt et al., 2016; Liu et al., 2018; Li et al., 2019). Although these studies were homogeneous in terms of patient inclusion, they used different cardiotoxicity definitions, different endpoints, and the rationale behind selected genes in each study. Two studies were investigating SNPs that have earlier evidence of significant association with AIC (Vulsteke et al., 2015; Hertz et al., 2016), while two else explored genes active in two mechanistic pathways of cardiotoxicity (Reinbolt et al., 2016; Liu et al., 2018). **Table 1** summarizes the main characteristics of the design and outcomes of these studies. Among the SNPs selected and studied, five associations were found to be statistically significant; rs246221 in *ABCC1*, rs1045642 in *ABCB1*, rs1056892 in *CBR3*, rs10838611 in *ATG13*, and *UGT2B7* -161. One variant in *CBR3* showed conflicting results with Reinbolt and colleagues' showing no significant association with cardiac failure, while Hertz and colleagues showed that it is associated with a significant increase in the risk of reducing the ejaculation fraction to less than 55%. Each of these two studies used a different definition of cardiotoxicity and followed patients for different periods (Hertz et al., 2016; Reinbolt et al., 2016). This highlights one of the major reasons for the heterogeneity in pharmacogenomic studies outcomes and the difficulty in reaching consensus.

**Table 1:** PGx studies of anthracyclines cardiotoxicity (AIC) in breast cancer.

Sample	Anthracycline (dose)	Follow-up (year)	Cardiotoxicity definition	Rational of gene selection	Studied Genes*	SNPs found to be significantly associated in multivariable analysis	Reference
**877 early BC patients**	Epi (100 mg/m^2^)	Median 3.62	- Asymptomatic decrease of LVEF > 10% - cardiac failure grade 3–5	-10 SNPs in 6 genes were previously found in association with AIC -11 SNPs related to treatment outcome	*ABCC1* *ABCC2* *CYBA* *NCF4* *RAC2* *SLC28A3*	CT *at* rs246221 in *ABCC1* is associated with LVEF decline >10%, (p=0.02)	(Vulsteke et al., 2015)
**166 BC patients suspected to have AIC**	Dox (accumulative 240 mg/m^2^)	At least 12 months since receiving the anthracycline.	Systolic dysfunction defined by EF <55%	Variants previously reported to be associated with AIC or that had mechanistic rationale	*ABCB1* *ABCB4* *CBR3* *CYP3A4* *NCF4* *RAC2* *SLC28A3* *TOP2B*	- rs1045642 in *ABCB1* Has protective effect (p=0.049) - rs1056892 in *CBR3* with increased risk of EF < 55% (p=0.002)	(Hertz et al., 2016)
**162 BC patients**	N/A	A retrospective study that collected data from 9 years span	Cardiomyopathy defined by a drop in EF to <50% or >15% decrease from pre-therapy and developing a new arrhythmia or MI after therapy	Genes in carbonyl reductase pathways	*CBR1* *CBR3*	No association was found in the studied SNPs	(Reinbolt et al., 2016)
**147 triple-negative BC patients**	N/A	A retrospective study that collected data from 7 years span	Early-onset cardiac events (any change in the ECG following any of the first 4 cycles of therapy)	Selected SNPs related to autophagy	*ATG5* *ATG7* *ATG12* *ATG13* *ATM* *CASP3* *CRYAB* *MAP1LC-3B* *STMN1*	rs10838611 in *ATG13* with early ECG abnormalities	(Liu et al., 2018)
**427 BC patients**	Epi (100 mg/m^2^)	12 months	Absolute decline in LVEF at least 10 points from baseline or to less than 53%, heart failure, coronary syndrome, or arrhythmias	The selected gene is involved in anthracyclines metabolism by glucuronidation	*UGT2B7*	T allele *UGT2B7* -161 with decreased risk of ACT (P = 0.004)	(Li et al., 2019)

AIC, anthracycline-induced cardiotoxicity; BC, breast cancer; Epi, epirubicin; Dox, doxorubicin; EF, ejection fraction; ECG, elecrtocardiography; LVEF, left ventricular ejection fraction.

*The mentioned genes were not fully covered in these studies, only specific polymorphisms in these genes were tested.

Two more studies recruited BC patients exclusively but used the GWAS approach. Again, these studies used a different endpoint for cardiotoxicity, and the utilized microarrays platforms were different. In the first and the larger study with more than 6,000 patients, rs28714259, which lies in an intergenic region of the genome, was the most significantly associated SNP with cardiotoxicity (Schneider et al., 2017). The second study, with only 154 patients, found a significant association of chronic AIC with the electron transfer flavoprotein beta (*EFTB*) gene (Ruiz-Pinto et al., 2018). None of these associations were verified in confirmatory studies or characterized functionally yet.

In summary, there is no substantial evidence for any variant association with AIC in BC. The evidence supporting the carbonyl reductase gene *CBR3* is amongst the strongest, given the reductase contribution in catalyzing the cardiotoxic metabolites of anthracyclines (Reinbolt et al., 2016). More research can confirm or disprove this association as well as the other reported singleton associations with genes in anthracyclines or cardiotoxicity pathways.

### PGx Studies on Taxanes Induced Neuropathy

Neurotoxicity induced by paclitaxel has been reported from several studies in ovarian cancer patients; however, in these cases, paclitaxel is usually given with platinum drugs. Platinum drugs might also induce neuropathy. Accordingly, breast cancer cohorts were other neurotoxic agents are rarely used, are more suitable to investigate genetic biomarkers of TIN (Abraham et al., 2014).

Six candidate-gene studies were found in the literature that investigated the PGx biomarkers for TIN in BC (Sucheston et al., 2011; Hertz et al., 2013; Abraham et al., 2014; Eckhoff et al., 2015; Kus et al., 2016; Lam et al., 2016; Tanabe et al., 2017). Most of the targeted genes where either in the metabolic pathways of taxanes or have been found associated with TIN in previous work.

Notably, three studies found a significant association between *CYP2C8* low function alleles and the development of neurotoxicity following taxane use (Hertz et al., 2013; Abraham et al., 2014; Lam et al., 2016). The enzyme encoded by *CYP2C8* is the primary cytochrome P450 metabolizer of paclitaxel (Hertz et al., 2014). Nevertheless, the association between *CYP2C8* and taxanes is classified as a level 3 clinical annotation by the PharmGKB database (www.pharmgkb.org). Level 3 designates variant-drug associations that are either found in single studies and are not replicated or are lacking clear evidence. We propose this association as a proper candidate for further investigation, mainly because one independent GWAS found the genetic variant with the highest association to neurotoxicity is located in *CYP2C8* (Hertz et al., 2014).

Another significant association in *ABCB1* was retrieved from three different studies. Two of these variants, rs1045642 and rs1128503, were found to be associated with an increased risk of neuropathy (Kus et al., 2016; Tanabe et al., 2017). In contrast, rs3213619 was associated with a decreased risk of sensory neuropathy (Abraham et al., 2014). Regardless of these contradictory results, there is accumulating evidence on the involvement of *ABCB1* in developing TIN that worth further evaluation. *ABCB1* encodes a vital transporter that has been repeatedly related to response and resistance to chemotherapy but its contribution to toxicity development is not well studied (Hodges et al., 2011).

Sucheston and coworkers investigated two non-pharmacokinetic related genes that are active in one of the DNA repair pathways. DNA repair is thought to be one of the mechanisms involved in the development of TIN. Interestingly, a significant association was found between low activity alleles in *FANCD2* and the development of TIN (Sucheston et al., 2011). However, this finding was not replicated yet in any other cohort. The details of all targeted-gene studies related to TIN retrieved from the literature review are summarized in **Table 2**.

**Table 2:** PGx studies of Taxane-Induced Neuropathy (TIN).

Sample	Taxane used (Dose)	Neuropathy definition	Rational of gene selection	Studied genes**	SNPs found to be significantly associated in multivariable analysis	Reference
**888 BC patients**	Paclitaxel	- Motor coordination, pain, and myalgias. - FACT-TAX neurotoxicity score	Genes in Fanconi anemia/BRCA1 pathway which is known to be effective in DNA repair pathway	*BRCA1* *FANCD2*	*FANCD2* low expression alleles and haplotypes (rs7648104, rs7637888, rs6786638, rs6442150) are associated with TIN (p < 0.001)	(Sucheston et al., 2011)
**411 BC patients**	Paclitaxel (80–90 mg/m^2^/week Or 175 mg/m^2^/2 or 3 weeks)	Grade ≥ 2 neuropathy during therapy	One decreased-activity haplotype in a gene of known activity in paclitaxel metabolism	CYP2C8*3	CYP2C8*3 variant is significantly associated with increased risk of neuropathy (p=0.031)	(Hertz et al., 2013)
**1303 BC patients**	Paclitaxel (175 mg/m2)	- Grade ≥ 2 neuropathy during therapy - Time to	Previously studied candidate SNPs selected from 17 previous studies	73 SNPs in 50 genes	*CYP2C8* (rs1058930) *ABCB1* (rs3213619) is associated with a decreased risk of TRSN (p=0.004). *TUBB2A* (rs9501929) is associated with increased risk of TRSN (p=0.005) *EPHA6* (rs301927) is associated with increased risk of TRSN (p=0.01)	(Abraham et al., 2014)
**150 BC patients**	docetaxel 75 to 100 mg/m^2^	Induced peripheral neuropathy	Genes known previously to be associated with TIN or in docetaxel metabolic pathway	*ABCB1* *ATP7A* *CHST3* *CYP3A5* *ERCC1* *GSTP1* *NAT2* *SLCO1B3* *SLC10A2*	*GSTP1* rs1138272 C/T or T/T genotype (P=0.01)	(Eckhoff et al., 2015)
**219 BC patients**	Paclitaxel (175 mg/m^2^ every 3 weeks for four cycles) Or Paclitaxel 80 mg/m^2^ weekly for 12 cycles) and Docetaxel (100 mg/m^2^ for four cycles)	Neurotoxicity symptoms were checked before each cycle	Genes in transport, metabolism, and pharmacodynamics of taxanes	*ABCB1* *CYP2C8* *CYP3A4* *ERCC1* *ERCC2* *FGFR4* *TP53* *ERBB2*	*ABCB1* rs1045642 was associated with a higher risk of grade ≥ 2 neurotoxicity (p=0.017)	(Kus et al., 2016)
**188 women with Her2-negative metastatic BC**	Paclitaxel (90 mg/m2)	- Grade ≥ 1 neurotoxicity. - Cumulative dose until first dose reduction due to neurotoxicity.	SNPs that were previously found to be associated with neurotoxicity	CYP2C8*3 CYP3A4*22 *FGD4* *EPHA5* *TUBB2A*	- CYP2C8*3 received a lower cumulative dose until the development of Grade≥1 neurotoxicity. - CYP2C8*3 is a significant marker for neurotoxicity (in multivariate analysis) (p=0.045) - *FGD4* c.2044-236 G > A is associated with a lower cumulative dose before the development of Grade≥1 neurotoxicity.	(Lam et al., 2016)
**127 BC patients**	Paclitaxel (80 mg/m2) weekly for 12 cycles.	Peripheral neuropathy was evaluated at baseline, at week 7, within 7 weeks after the final paclitaxel dose and after one year.	SNPs that have previously shown an association with paclitaxel-induced peripheral neuropathy.	*SLCO1B3* *CYP2C8* *ABCB1*	*- ABCB1* rs1128503 was significantly associated with grade ≥ 2 only in patients ≥ 60 years (p=0.027). - The multivariable analysis concluded a significant risk factor of this variant and age in grade ≥ 2 peripheral neuropathy (p=0.005).	(Tanabe et al., 2017)

TRSN, taxane related sensory neuropathy; FACT-TAX, Functional Assessment of Cancer Therapy-Taxane

**The mentioned genes were not fully covered in these studies, only specific polymorphisms in these genes were tested.

Two GWAS studies were found to investigate TIN biomarkers across the genome. The first pointed to a novel biomarker in the congenital peripheral neuropathy gene *FGD4* as an effector in the onset of neuropathy, besides other two novel probable associations (Baldwin et al., 2012). While the other concluded that CYP2C8 poor metabolizers are at higher risk of developing grade ≥2 neurotoxicity following paclitaxel treatment (Hertz et al., 2014), which was in concordance with the targeted-gene studies (Hertz et al., 2013; Abraham et al., 2014; Lam et al., 2016).

To summarize, *CYP2C8*, encoding one of the main metabolizing enzymes of paclitaxel, warrants further validation as a predictor for paclitaxel-induced neuropathy. This association is one of the few associations which were retrieved in both the targeted gene design and the presumption-free design (GWAS) studies. While the contribution of the transporter encoding gene *ABCB1* is another suitable candidate for studying. This very important pharmacogene, according to PharmGKB classification, warrants further evaluation in the context of neurotoxicity and side effects on other systems, as described in the following section.

### PGx Studies of Hematological Toxicity of BC Chemotherapies

Hematological toxicity is the most common adverse event encountered with cytotoxic agents. Anemia, neutropenia, and thrombocytopenia are all different forms of myeloid toxicity. These “cytopenias” are the most encountered dose-limiting chemotherapy-induced toxicities. This manifestation can be explained by the rapid mitotic rate of myeloid cells, which makes them vulnerable targets of cytotoxic agents. While lymphopenia is less common, though it is severe toxicity that increases the risk of life-threatening infections (Kurtin, 2012).

Reports show that around 50% of BC patients develop neutropenia of any grade during chemotherapy, while 20% suffer from a more severe form, which is febrile neutropenia requiring hospitalization. Interindividual differences in risk of cytopenia and febrile neutropenia are frequently encountered, of which some may be explained by genetic variation (Bidadi et al., 2018). Since many of the cytotoxic agents in BC are given within different combinations, and most of these can induce hematological toxicity, it has been challenging to confirm any specific association between a gene and a single agent, although a large number of studies addressing this issue. **Table S1** (supplementary) summarizes original papers in the literature that investigated hematological toxicity during BC chemotherapy classified according to the regimen used.

*ABCB1*, which encodes for a transporter and efflux protein, was the most commonly studied candidate gene concerning hematological toxicity (Chang et al., 2009; Cizmarikova et al., 2010; Rizzo et al., 2010; Ji et al., 2012; Kim et al., 2012; Chaturvedi et al., 2013; Yao et al., 2014; Chaturvedi et al., 2015; Faraji et al., 2016; Tsuji et al., 2016; Angelini et al., 2017). Most of these studies did not find a significant association between *ABCB1* variants and hematological toxicity induced by any cytotoxic regimen except for four studies (Kim et al., 2012; Chaturvedi et al., 2013; Voon et al., 2013; Angelini et al., 2017). However, the association found in these studies resulted from the univariate analysis where other genetic or non-genetic variants were not considered. Moreover, these four studies are heterogeneous in terms of the studied cytotoxic agent and the genotyped variants in *ABCB1*.

The association of *CYP3A5* (rs776746) with hematological toxicity was analyzed in three studies (Tang et al., 2013; Tsuji et al., 2016; Angelini et al., 2017) but in different contexts. No significant association between the patient's genotype at this variant and Taxanes-induced hematological toxicity was detected (Angelini et al., 2017). However, a significant protective effect of the G allele of rs776746 was found when neutropenia following any cycle of AC was evaluated (Tang et al., 2013). An observation that was not replicated when neutropenia during the first cycle only of AC therapy was evaluated (Tsuji et al., 2016). This illustrates how contradictory data can originate from evaluating the genotype-toxicity associations in different chemotherapy regimens or due to variability in data collection time points.

CYP2B6*6 was found to have a protective effect from high grades neutropenia (Tsuji et al., 2016). However, the same haplotype, determined by rs3745274, did not show any significant association when the need for dose reduction, erythropoietin use, transfusion, or iron supplementation was considered as the studied outcomes (Haroun et al., 2015).

Similarly, conflicting results are seen in the association between *SOD2* variants and hematological toxicity. *SOD2* encodes for a critical enzyme in the oxidative stress pathway and is thought of as an essential component in defense against reactive oxygen species (ROS). Accordingly, it was hypothesized that variants causing low enzyme activity would increase the accumulation of ROS in healthy tissues leading to toxic side effects, including hematological toxicity. This hypothesis was supported by a relatively large study that included 458 BC patients treated with CAF or CMF regimens. Patients carrying the low activity alleles (rs4880) had double the risk of grade ≥ 3 neutropenia or leucopenia compared to wild type carriers (Yao et al., 2010). The association was not retrieved when the same variant was analyzed in 153 Chinese BC patients treated with TA, TAC, or FAC (Ji et al., 2012). However, the effect of the oxidative stress pathway is studied more comprehensively in the context of BC treatment response and puts this pathway as a strong candidate for further investigation in the context of treatment toxicity.

Glutathione-S-transferases (GSTs) are a group of phase II metabolic conjugation enzymes that are involved in the metabolism of chemotherapy agents, including anthracyclines. Several studies have evaluated the association between variants in genes coding for GSTs and response to chemotherapy in BC, but few have evaluated drug toxicities. Tulsyan and coworkers focused on three GST members, *GSTM1*, *GSTP1*, and *GSTT1,* and found one significant association between *GSTP1* rs1695 and grade ≥2 anemia. However, this association was found in a univariate analysis and was not retrieved when multivariate analysis was applied in the same study (Tulsyan et al., 2013). No significant association between variants in the same genes and hematological toxicity was detected by Tsuji and colleagues (Tsuji et al., 2016).

*CBR1 and CBR3*, which encode essential enzymes (i.e., carbonyl reductases) in the metabolic pathway of anthracyclines, have been studied in association with anthracycline cardiotoxicity as shown previously. As hematological toxicity is another significant dose-limiting toxicity for anthracyclines, both genes were also evaluated for association with this adverse event (Fan et al., 2008; Voon et al., 2013). Only one association was detected between CBR3 rs8133052, and low blood counts reported early in the anthracycline course (Fan et al., 2008).

Finally*, SLC22A16*, a solute carrier active in anthracyclines transportation, is another proposed contributor in anthracyclines-induced hemato-toxicity (Voon et al., 2013; Faraji et al., 2016). However, this association was not supported by any statistically significant evidence.

Apart from the previously mentioned genes, several other genes were investigated for association with hematological toxicity but each in a single cohort (Rizzo et al., 2010; Tang et al., 2013; Yao et al., 2014; Chaturvedi et al., 2015; Tsuji et al., 2016; Angelini et al., 2017). In conclusion, the targeted gene approach failed to lead to a hematological toxicity biomarker during BC chemotherapy despite the severity of these events and their life-threatening effects.

However, a recent GWAS that covered more than 325,000 variants located in 468 genes investigated biomarkers of hematology toxicity in 3,754 BC patients found significantly associated variants in *HMMR* locus. Among these patients, more than 40% developed grade ≥3 leukopenia, neutropenia, or febrile neutropenia during cycles of CEF, which is within the reported range of hematological toxicities in breast cancer. The *HMMR* was further investigated in the same study to find out the mechanism of its action on hematological suppressive pathways. Functional studies revealed that the SNPs in this gene act as trans expression quantitative trait loci (Trans-eQTLs) that affect the expression of another gene, namely *TNFSF13B*, which is a modulator of chemotherapy sensitivity. The findings of this study demonstrate the complexity of chemotherapy-induced hematological toxicity pathways (Bidadi et al., 2018).

To recap, the transporter proteins, especially the one encoded by *ABCB1*, and the cytochrome P450 metabolizing enzymes have been frequently investigated without conclusive evidence on any hematological toxicity biomarker. Even though more studies on larger cohorts treated with unified regimens that utilize specific toxicity indicators are needed to elucidate biomarkers on any of these genes.

### PGx Studies of Other Systemic BC Chemotherapy Toxicities

Other systemic toxicities are commonly encountered during BC chemotherapy. The most frequently reported are nausea and vomiting, diarrhea or constipation, pain, arm swelling, and breast skin irritation (Friese et al., 2017). High susceptibility to infections leading to hospitalization is another severe systemic side effect. A common practice when a high grade of these events occurs is to delay one or more doses of the planned regimen or to reduce the given dose below the recommended one. Nevertheless, these practices put the patient under the risk of not getting the full benefit of treatment (Bray et al., 2010).

Multiple pharmacogenomic studies have evaluated different toxicities during BC chemotherapy but were not consistent in their selected outcome, their selected genes, and the used chemotherapeutic regimens. **Table 3** lists toxicity pharmacogenetic studies in BC with different endpoints.

**Table 3:** PGx of miscellaneous toxicities in BC associated with different chemotherapeutic regimens.

Regimen	Sample	Toxicity definition	Rational of gene selection	Studied genes**	Significant genetic associations	Reference
AC	230 Early-stage BC patients*	- Need for dose delay - Need for dose reduction - Inability to complete the planned course.	Genes involved in the metabolism or transport of AC.	*ABCB1* *CYP2B6* *CYP2C19* *CYP2C9* *CYP3A5* *SLC22A16*	- CYP2B6*5 and *2 associated with a greater incidence of dose delay - *SLC22A16* T1226C was associated with a greater incidence of dose delay and leucopenia	(Bray et al., 2010)
AC	227 Early-stage BC patients*	- Need for dose delay	Prior data for the effect of *NQO1* on the outcomes of anthracycline regimens. *NQO2* was added due to its functional homology to *NQO1*.	*NQO1* *NQO2*	Carriers of the minor allele at *NQO1* rs1800566 showed lower frequency of dose delay (P=0.01)	(Jamieson and Boddy, 2011)
AC	822 BC patients	Grade III gastrointestinal toxicity	variants in Pharmacokinetic genes for CP and DOXO	*ABCB1* *ABCC1* *ALDH1A1*	None	(Yao et al., 2014)
AC	265 Early-stage BC patients*	- Grade III infections (infections that lead to hospitalization). - Other lower grade infections “any infection.”	Two genes that are known to have a function in the immune response	*CD95* *MBL2*	*CD95* (rs2234767) minor allele is significantly associated with grade III infections (p=0.048) and any infection (p=0.047) (but not significant when corrected for multiple testing). MBL2-221 (rs7096206_ minor allele is significantly associated with grade III infection (p=0.048)	(Jamieson et al., 2017)
Multiple regimens (CP-based)	403 BC patients (184 cases; i.e. suffered from adverse reactions and 219 controls)	- Grade≥ III gastrointestinal toxicity	Tag and functional SNPs in 13 genes involved in activation, detoxification, or transportation of CP	*ABCC2* *ABCC4* *ALDH1A1 ALDH3A* *CYP2B6* *CYP2C9 CYP2C19 CYP3A4* *CYP3A5* *GSTA1* *GSTM1* *GSTP1* *GSTT1*	ABCC4 (rs9561778)	(Low et al., 2009)
Multiple regimens	50 primary BC patients	Any kind and grade of toxicity	SNPs in genes related to folate metabolism pathway	*ABCB1* *MTHFR* *TYMS*	-Allele T at *MTHFR* (rs1801131) (p=0.029) -Allele G (Argenin) at P53 (rs1042522) (p=0.018)	(Henríquez-Hernández et al., 2010)
Taxane-based	152 BC patients	Gastrointestinal, cutaneous, asthenia, mucositis, neurotoxicity, and others.	Genes on the metabolic pathway of taxanes	*ABCB1* *CYP3A4* *CYP3A5*	None	(Angelini et al., 2017)
Taxane-based	120 BC patients	Any kind and grade of toxicity	Genes involved in taxanes pathways or implied in DNA repair or ROS metabolism	*ABCB1* *ABCC2* *ABCG2* *CBR3* *CYP1B1* *CYP2C8* *CYP3A4* *CYP3A5* *ERCC1* *ERCC2* *GSTM3* *GSTP1* *MTHFR* *NOS3* *NQO1* *TP53* *UGT1A1* *UGT1A9* *XPC* *XRCC1*	Docetaxel: *ERCC1* (rs3212986) with mucositis grade ≥2 and CYP3A4*1B (rs2740574) with IRR grade ≥2 (p < 0.01) Paclitaxel: *CYP2C8* (rs1113129) and *CYP2C8 *(rs1934951) with anemia grade ≥2 and *ERCC1* rs3212986 with neuropathy grade ≥2	(Bosó et al., 2014)
Taxane-based	95 BC patients	- Neurological toxicity **-** Hypersensitivity reactions	Paclitaxel metabolism pathway	*CYP2C8* *CYP1B1* *ABCB1*	CYP1B1* 1 with hypersensitivity reaction (p < 0.0001)	(Rizzo et al., 2010)
AC/TC	155 early stage BC patients	Febrile neutropenia occurrence	Genes involved in the metabolism or transportation of docetaxel	1239 SNP in 183 gene	Haplotype TT at *SLCO1A2* (rs4762699) and (rs2857468) (p < 0.0001) in patients in taxane arm	(Callens et al., 2015)
AC-T	59 BC patients	- Fever (temperature ≥38.5 or >38 twice 2 h apart) or infection with leucopenia or neutropenia - The need for antidiarrhea medication prescription - Edema - Plural effusion	Genes involved in the metabolism or transportation of docetaxel	*ABCB1* *CYP3A4* *CYP3A5*	None	(Tsai et al., 2009)

AC, anthracycline and cyclophosphamide; AC-T, anthracycline and cyclophosphamide followed by taxane; CP, cyclophosphamide; FN, febrile neutropenia; TC, taxane and cyclophosphamide.

*The three studies were applied on the same cohort.

**The mentioned genes were not fully covered in these studies, only specific polymorphisms in these genes were tested.

Some researchers opted to measure the frequency of dose delay, adjustment, or treatment cessation due to treatment toxicities, while others chose the endpoint to be any type or grade of toxicity. The third group of studies focused on specific symptoms, like gastrointestinal symptoms, or an event, like infection, at a selected grade. However, most were investigating a limited number of biomarkers in genes encoding the metabolizing cytochrome p450 enzymes, or ATP-binding cassette family members active in cytotoxic agents' efflux and transport. The heterogeneity of these studies in design precludes comparing their outcomes.

One GWAS study was conducted to investigate genomic biomarkers of chemotherapy-induced alopecia in BC patients. In this work, Chung and coworkers compared 303 BC patients who suffered from docetaxel-induced alopecia with 880 BC patients who did not. They found one significant SNP in the calcium channel voltage-dependent subunit beta 4 (*CACNB4*), besides several other suggestive SNPs.

Rigorous study design, which takes into account the incidence rate of the systemic side effect, chooses a representative sample size, and uses a technique that can cover all the genes involved in the pathways under focus can lead to strong associations. Further confirmatory studies of the reported associations in BC chemotherapy systemic side effects are needed.

### PGx Studies of Capecitabine Toxicities

Due to the high frequency of HFS among capecitabine treated patients, several pharmacogenomic studies have investigated biomarkers that might be used to predict susceptible patients. Variants in *SPRY2*, *MACF1*, and *DPYD* were found to be significant susceptibility biomarkers for HFS in a large GWAS study that included 138 BC patients, among other types of tumors (Yap et al., 2017).

Two studies investigating capecitabine toxicity biomarkers were found to have exclusively recruited BC patients. In the first and older one by Largillier and coworkers, three genes (*TS*, *MTHFR*, and *DPYD*) active in the pharmacodynamics of capecitabine were investigated. In this study, among 105 advanced BC patients, 17.9% suffered from grade 3 or 4 of any type of toxicity after the first capecitabin cycle. In the third cycle, the rates of nausea and vomiting, hematological and gastrointestinal toxicities reduced in contrast to the rates of HFS, which increased from 5.2% in the first cycle to 9.9% in the third cycle. However, analysis of the genotypes at SNPs in the selected genes did not give any significant association with any type of toxicity (Largillier et al., 2006).

In the other more recent study by Etienne-Grimaldi and colleagues, all exons of *DPYD* were examined, and two variants (DPYD*2A and D949V) were found to be significantly associated with an increased risk of grade ≥ 3 hematological, digestive, or neurological toxicities (p=0.041). These findings were in concordance to previous evidence that suggested *DPYD* and its product enzyme dihydropyrimidine dehydrogenase (DPD) as a strong predictor of capecitabine toxicity (Etienne-Grimaldi et al., 2017).

Although both studies have examined *DPYD* against the same toxicities, their outcomes are not comparable and cannot be considered as contradictory. While Etienne-Grimaldi and colleagues used next-generation sequencing to cover all the coding regions of *DPYD,* Largillier and colleagues used a simple genotyping technique to genotype one common low activity variant. Hence, the used technique and its coverage are crucial determinants of any pharmacogenomic study power and outcomes.

Recently, the Dutch pharmacogenomics working group (DPWG) published guidelines regarding *DPYD* testing prior to the initial dose of capecitabine and 5-FU in any kind of cancer, including breast cancer. The DPWG concluded that four variants, DPYD*2A (c.1905+1G > A, IVS14+1G > A) and DPYD*13 (c.2846A > T and c.1236G > A), have sufficient evidence for implementation in clinical practice. The recommendations include avoiding the use of fluoropyrimidines in cases where the gene activity score equals zero and reducing the initial dose to 50% of the standard dose in cases were activity score is 1 to 1.5. The same guidelines include a gene activity score-explaining Table (Lunenburg et al., 2020).

### PGx Studies of Platinum Compounds Toxicities

Due to the limited use of platinum compounds in BC compared to other types of solid tumors, especially lung, gastric, and colorectal cancer, few studies have evaluated the toxicity of these compounds in BC patients or have investigated any pharmacogenomic biomarkers for these toxicities.

One umbrella systematic-review, in which systematic reviews and meta-analysis were analyzed and validated, failed to find any significant pharmacogenomic biomarkers of platinum compound toxicities during their use for any kind of tumor (Campbell et al., 2016).

The recent evidence of the value of these compounds in BC warrants investigating the expected toxicities and their candidate biomarkers. A suggested gene to investigate is *ERCC1*, which was proposed as a predictor of platinum compounds associated nephrotoxicity (Tzvetkov et al., 2011), with conflicting evidence (Khrunin et al., 2012). Other proposed candidate genes include those active in platinum compound pathways, comprehensively described in the PharmGKB database (Platinum Pathway), of which some were explored for association with response to these compounds rather than toxicity. However, the same genes might be good candidates for exploring their association with developing side effects and toxicities.

### PGx Studies of Long-Term Side Effects

With the current improvement in cure and survival rates among BC patients, late-onset sequelae of chemotherapy require increased attention. These delayed effects include late-onset anthracycline-induced cardiac toxicity, secondary cancers (particularly acute myeloid leukemia and myelodysplastic syndrome), early menopause, infertility, and sexual dysfunction. Besides, contradictory data about chemotherapy long-term effects on cognitive function. However, the incidence of delayed events might be under-estimated because of missing data from patients who died or relapsed (Azim et al., 2011).

The suggested risk factors of late-onset side effects are accumulated dose, age, exposure to radiation therapy, and co-morbidities. Pharmacogenomic contribution in delayed side effects is not studied except for a few studies conducted on cardiac toxicity induced by anthracyclines, which we have discussed earlier. Given their increasing numbers, large studies of BC survivors can produce data about PGx biomarkers disposing of delayed side effects.

## Conclusion and Future Directions

Chemotherapy is an indispensable component of systemic therapy in many cases of BC. Reducing chemotherapy side effects can improve patients' quality of life and prevent treatment cessation by practitioners or treatment refusal by patients.

Pharmacogenomic studies are conducted to investigate biomarkers that can predict the toxicity or efficacy of chemotherapies. A relatively limited number of pharmacogenomic studies have been conducted to investigate these biomarkers in BC with inadequate accumulated evidence to warrant specific dosing or regimen guidelines by regulatory bodies. Apart from the guidelines regarding *DPYD* testing prior to fluoropyrimidine use, there are no other guidelines related to other chemotherapeutic agents used in BC.

The primary outcomes of the reviewed pharmacogenomic studies include suggesting *CBR3* as a candidate biomarker, which needs further investigation for anthracycline-induced toxicities, including cardiac and hematological toxicities. Besides, repeated evidence pointing to *CYP2C8* as a biomarker for TIN induced by paclitaxel. Variations in *ABCB1* were also proposed as susceptibility biomarkers for paclitaxel-induced toxicity with contradictory evidence. Variants at the latter gene are also suggested as biomarkers for hematological toxicity caused by any combination of cytotoxic agents. Moreover, many other genes were found, either in a single study or a few numbers of studies, to be probably associated with other toxicities.

The notable limitations in reaching a consensus from the reviewed studies were (1) heterogeneity in defining toxicity or choosing the toxicity endpoint, (2) following patients for different periods, (3) inconsistency in the compared chemotherapy regimens, and (4) inconsistency of evaluated genes or even in the genotyped variants from the same gene. There is a need to apply confirmatory studies for the detected associations; furthermore, functional studies would provide strong evidence of the suggested biomarkers.

The importance of pharmacogenomic research of chemotherapy toxicity in BC might be undermined by the fact that many chemotherapy agents are available for these patients. However, some favorable regimens are commonly used and are associated with severe side effects. Besides, many patients need multiple lines of therapies due to recurrence or relapse, so they will be endangered by encountering more adverse events.

There are opportunities in pharmacogenomic research of BC chemotherapy toxicity. Long lists of gene-drug and gene-toxicity pairs have been accumulated from previous research that worth reinvestigation and reevaluation. However, future research should be designed to avoid the limitations of previous studies through stringent designs that reduce the magnitude of selection and information bias. The selection of statistical tests to reduce false positives is another meaningful aspect to consider. With the advent of high throughput techniques, such as next-generation sequencing, at affordable prices, researchers are expected to utilize these advances rather than using the same old genotyping techniques that cover a fewer number of variants. Several levels of “omics” as proteomics, transcriptomics, metabolomics with the unparalleled advent in big data analysis all should be integrated into the investigation of biomarkers of toxicity.

Finally, association studies are just the tip of the iceberg. The implementation of their findings into the clinic needs enormous steps of validation. Researchers are expected to prove the cost-effectiveness of any pharmacogenomic finding before moving it from the research lab into clinical practice in the form of personalized and precision approaches.

## Author Contributions

ZA-M data curation, analysis and writing the original draft. GP: review of analysis, and manuscript review. BA: conceptualization, manuscript review and editing, and funding acquisition.

## Funding

This work was supported by the United Arab Emirates University funding grants (31R091 and 31M341).

## Conflict of Interest

The authors declare that the research was conducted in the absence of any commercial or financial relationships that could be construed as a potential conflict of interest.
