# Supplementary material for: Toxicity and Pharmacogenomic Biomarkers in Breast Cancer Chemotherapy
Source: Front Pharmacol. 2020 Apr 15;11:445. doi: 10.3389/fphar.2020.00445 (PMC7174767; doi:10.3389/fphar.2020.00445)
Supplement: Supplementary file 1 [file Table_1.docx]

Supplementary Material

**Table S1: Hematological toxicity of chemotherapy regimens used in BC**

| Regimen | Sample | Toxicity^1^ | Rational of gene selection | Studied Genes | Genotyped SNPs^2^ | Significant genetic associations^3^ | Significant non-genetic associations^3^ | Reference |
| --- | --- | --- | --- | --- | --- | --- | --- | --- |
| AC | 311 Chinese BC patients | grade IV neutropenia ANC<500/µl | Genes in metabolic pathways of DOX and CP | *PXR*  *CYP3A4*  *CYP3A5* | rs1464603  rs2276707  rs2242480  rs776746, | CYP3A5*3 (rs776746)  G allele has protective effect (p=0.023) | BMI (p=0.014)  Baseline ANC (0.02)  GCSF need during therapy (p<0.001) | Tang et al 2012 |
| AC | 822 BC patients from North America  (83% European American, 8% African American, 5% Asian American, 4% other) | Grade III or IV hematological toxicity | variants in Pharmacokinetic genes for DOX and CP | *ABCB1*  *ABCC1*  *ALDH1A1* | 78 tagSNPs | Haplotype of the minor alleles at *ALDH1A1*  (rs3764435, rs168351) is associated with increased risk of toxicity (p=0.03) | None | Yao et al 2014 |
| AC | 100 Japanese BC patients | grade IV neutropenia based on the lowest count of neutrophils during the first cycle | Genes in metabolic pathways of DOX and CP | *ABCB1*  *CYP2B6*  *CYP3A5*  *GSTM1*  *GSTP1*  *ERCC1* | rs1128503  rs2032582  rs1045462  rs3745274  rs2279343  rs3211371  rs776746  M1/T1 null  rs1695  rs11615  rs3212986 | *CYP2B6*6 with protective effect* (p=0.025)  *ERCC1* (rs11615) with  Increased risk (p=0.020) | BMI  baseline WBC count | Tsuji et al 2016 |
| FAC/FEC | 207 BC patients from north India ^#^ | Grade II-IV hematological toxicity | Gene active in transportation of cytotoxic drugs | *ABCB1* | rs1128503  rs2032582  rs1045642 | ABCB1 (rs1128503)  T allele is associated with increased risk (p=0.049) | None | Chaturvedi et al 2013 |
| FAC/FEC | 207 BC patients from north India ^#^ | Highest grade of hematological toxicity | Genes in metabolic pathways of | *GSTM1*  *GSTT1*  *GSTP1* | Present/null  Present/null  rs1695 | *None* | Tumor stage | Tulsyan et al 2013 |
| FAC/FEC | 145 BC patients from Lebanon (toxicity data was collected from 45 only) | - Incidence of FN  - Need of CP dose reduction  - Need for GCSF  - Need for packed RBCs transfusion, iron supplements or erythropiotin | Variants involved in CP clearance | *CYP2B6* | rs2279343  rs3211371 rs3745274 | *None* | None | Haroun et al 2014 |
| CAF/CMF | 458 primary BC patients from North America  (88% non-Hispanic) | Grade III or IV neutropenia and leucopenia | Gene active in the oxidative stress pathway in which the selected variant will decrease its activity | *SOD2* | rs4880 | *SOD2* (rs4880) CC associated with half toxicity risk compared to TT (p=0.03) | None | Yao et al 2010 |
| FEC/FAC/FMC | 243 BC patients from India | Grade II-IV hematological toxicity | - A gene in folate pathway (MTHFR).  - NQO1 is thought to affect response to Dox/Epi | *MTHFR*  *NQO1* | rs1801133  rs1800566 | NQO1 (rs1800566) has protective effect from toxicity (p=0.027) | None | Chaturvedi et al 2015 |
| FAC / FEC / AC | 221 BC patients (111 included in toxicity analysis) | Hematological toxicities according to WHO criteria | The known role of the gene product as a transporter | ABCB1 (MDR1) | rs1045642 | None | None | Cizmarikova et al 2010 |
| Doxo-T | 99 Southeast Asian BC patients (Singapore) | -Lowest blood count documented within 21 days from Dox | Two genes from the Dox metabolic pathway | *CBR1*  *CBR3* | *Coding regions of both genes* | CBR3 minor allele at rs8133052 (p=0.018) | None | Fan et al 2008 |
| Doxo-T | 151 BC patients from Singapore (57% Chinese and 34% Malay) | Lowest counts after the first cycle of Doxo | Genes in metabolic pathway of Doxo | *AKR1C3*  *ABCB1*  *CBR3*  *SLC22A16* | Exon 5  rs2032582  rs4148350  rs2235047  rs8133052 rs1056892  rs6907567  rs12210538  rs723685 | AKR1C3 IVS4-212 GG is significantly associated with lower leucocytes counts (p=0.007)  ABCB1 (rs2235047) (IVS26+59 T>G) is associated with higher neutrophil and leucocyte counts. | Metastatic status  Histological grade | Voon et al 2012 |
| DOX-Based | 100 BC patients from Iran (50 in case group “had grade ≥ 3 neutropenia” & 50 in control group grade ≤ 2 neutropenia) | neutrophil count ≤ 1x109/L | Genes previously known to affect metabolism and transportation of anthracyclines | *ABCB1*  *SLC22A16* | rs10276036  rs12210538 | None | None | Faraji et al 2016 |
| Paclitaxel | 121 BC patients | Complete blood counts and toxicity assessment each cycle | Two SNPs in an efflux pump gene | *ABCB1* | rs1045642  rs2032582 | None | None | Chang et al 2009 |
| Taxane-based | 95 Caucasian BC patients | Hematological | Paclitaxel metabolism pathway | *ABCB1*  *CYP2C8*  *CYP1B1* | rs11285503  rs2032582  rs1045642  rs11572080  rs1058930  rs11572103  rs10509681  rs1056836 | None | None | Rizzo et al 2010 |
| Taxane-Based | 152 BC patients | Blood counts on days 14 and 20 of every cycle for hematological toxicity  - other studied toxicity: | Genes on the metabolic pathway of taxanes | *ABCB1*  *CYP3A4*  *CYP3A5* | rs1128503  rs1045642  rs2740574 (*1B)  rs776746 (*3) | *ABCB1* rs1045642 TT has a protective effect from toxicity (p=0.05) | None | Angelini et al 2017 |
| TAC/FAC | 153 BC patients: 89 patients on TA regimens, 10 patients on  TAC and 54 patients on FAC at neoAdj setting |  | Genes involved in anthracycline pathway and transportation | *ABCB1*  *SOD2*  *CAT*  *GSTT1*  *GSTP1*  *GSTM1* | rs1128503  rs2032582  rs1045642  rs4880  rs1001179  rs1695  Present/null  Present/null | None | None | Ji et al 2012 |
| AC-T | 218 BC patients with operable lymph node-positive BC from Korea | CBC at days 8 & 15 of T dose | Genes involved in metabolism, efflux and transport of docetaxel | *CYP3A5*  *ABCB1*  *ABCC2*  *SLCO1B3* | rs776746)  rs1045642  rs2032582  rs1128503  rs717620  rs2273697  rs3740066  rs4149117  rs7311358 | *ABCB1* (rs1045642)  T/T was significantly associated with neutropenia.  (*P* = 0.015) | Post-menopause (p=0.048) | Kim et al 2012 |
| Gemcitabine | 1678 BC | Grade III or IV neutropenia or leukopenia events during the first three cycles | Variants on genes identified from previous *in-vitro* GWAS study of gemcitabine toxicity and other genes from its metabolic pathway |  | 243 SNPs | *PIGB m*inor alleles at rs12050587 (p=0.03) and rs11636687 (p=0.03) | Age and BSA are significant predictors of neutropenia | Fasching et al 2017 |

**1** Toxicity; the selected toxicity endpoint which was used in statistical analysis **2** To insure homogeneity “rs numbers” are provided even if the original paper used other designation for the variants **3** Only significant associations are listed, non-significant or near significant associations were not mentioned

# Same cohort

AC; anthracycline and cyclophosphamide, BSA; body surface area, CP; cyclophosphamide, Dox; Doxorubicin, Epi; epirubicin, FAC; fluorouracil, anthracycline and cyclophosphamide, T; Docetaxel, FN; febrile neutropenia, 5-FU; 5-fluorouracil
